# *Cutibacterium acnes* Phylotyping and Antibiotic Resistance to Six Antibiotics: A Bulgarian Study

**DOI:** 10.3390/microorganisms13092185

**Published:** 2025-09-19

**Authors:** Lyudmila Boyanova, Georgi Dimitrov, Vessela Raykova, Kircho Patrikov, Raina Gergova, Rumyana Markovska

**Affiliations:** 1 Department of Medical Microbiology, Medical University of Sofia, 1431 Sofia, Bulgaria; l.boyanova@hotmail.com (L.B.); georgi.dimitrov.swy@gmail.com (G.D.); pumpi@abv.bg (V.R.); rtgergova@gmail.com (R.G.); 2 Clinic of Bone Pathology and General Orthopedics, Specialized Orthopedic University Hospital “Prof.B.Boychev”, Gorna Banya, 1614 Sofia, Bulgaria; k_patrikov@abv.bg

**Keywords:** *Cutibacterium acnes*, *Propionibacterium acnes*, acneic, non-acneic, orthopedic, phylotypes, antibiotic, antibiotic resistance, multidrug resistance

## Abstract

*Cutibacterium acnes* subspecies/phylotypes can cause infections requiring antibiotic therapy. Phylotyping of 73 (55 acneic and 18 non-acneic) *C. acnes* strains was performed, and antibiotic susceptibility was tested by E tests, breakpoint susceptibility test, or disk diffusion method. The dominant phylotype in both acneic and non-acneic strains was IA1 (56.2%). Phylotype II was >3-fold more frequent in non-acneic than acneic isolates. Resistance in acneic strains was >41% for clindamycin, 36.4% for tetracycline and 15.9% for levofloxacin, and that in non-acneic strains was >38% for clindamycin, 22.2% for tetracycline and 5.6% for levofloxacin. No strain was piperacillin/tazobactam or vancomycin resistant. Amoxicillin resistance was found in both acneic (5.4%) and non-acneic strains (11.1%), and was rare (1.8%) in phylotype I but higher (23.5%) in other strains. Double resistance was found in 32.6% of acneic and 22.2% of the non-acneic strains, and 9.3% of acneic strains displayed multidrug resistance. In conclusion, IA1 phylotype was dominant in both acneic and non-acneic strains, and type II was more frequent in non-acneic isolates. The detection (at >6%) of amoxicillin resistance represents a rare yet important finding. The presence of double/multidrug resistance strongly implies the need of susceptibility-guided therapy of the associated infections.

## 1. Introduction

*Cutibacterium acnes* is a Gram-positive aerotolerant anaerobic rod that is often detected in microbiology laboratories. *C. acnes* is a skin inhabitant of all adult people, and current knowledge reveals that *C. acnes* and *Staphylococcus epidermidis* together preserve skin homeostasis; however, skin dysbiosis can be affected by factors such as antibiotic use, and the ratio of *C. acnes* phylotypes can change, which may cause diseases such as acne vulgaris [1].

The species has three subspecies: *P. acnes* subsp. *acnes* (type I), *P. acnes* subsp. *defendens* (type II) and *P. acnes* subsp. *elongatum* (type 3) [2,3,4]. Different subspecies and phylotypes can be involved in the pathophysiology of different diseases such as acne vulgaris (mainly *C. acnes* subsp. *acnes* or phylotype I), sarcoidosis (probably *C. acnes* subsp. *acnes*), prostate cancer (*C. acnes* subsp. *defendens* or type II), infections of ventricular shunts, prosthetic joints, heart implants and different opportunistic infections (different phylotypes) [2,3,4].

Some *C. acnes* strains exhibit virulence factors associated with infections, such as fimbriae for adherence, CAMP factors, RoxP porphyrin, Christie–Atkins–Munch–Petersen factors, cytotoxicity, pTZC1 plasmid for multidrug resistance and biofilm production [3,4].

Therefore, *C. acnes* is important for human health as an inhabitant of healthy skin but can also be implicated in various diseases, many of which require antibiotic therapy.

*C. acnes* resistance to lincosamides, macrolides and tetracyclines usually results from single nucleotide polymorphisms (SNPs) in the ribosomal RNA genes, and fluoroquinolone resistance was linked to substitution in sequences of the DNA gyrase genes; different other mechanisms can lead to multidrug resistance [5,6]. However, there is scant information about beta-lactam non-susceptibility in the species.

The aim of the present study was to evaluate both phylotypes and susceptibility patterns of *C. acnes* strains to antibiotics in acneic and non-acneic strains isolated in our laboratory from October 2017 to April 2025.

## 2. Materials and Methods

The study was performed as part of the diagnostic activity of the laboratories in our Department of Medical Microbiology.

Seventy-three non-duplicate *C. acnes* strains were collected during the routine diagnostic practice of our laboratory. The strains from 55 patients with acne vulgaris and 18 patients with other diseases, mostly orthopedic infections, were evaluated. Patients’ characteristics and associated diseases are presented in Table 1.

According to the recommendations [7], the clinical specimens were taken after skin disinfection and deeply inserted into the Stuart anaerobic transport medium or were taken by swabs made of synthetic fibers (ESwab^®^, COPAN Diagnostics, Murrieta, CA, USA), which are appropriate for anaerobes.

The primers and PCR mix for *C. acnes* phylotyping were as described previously in the article of Barnard et al. [8].

The clinical samples were plated onto Anaerobe basal agar (Oxoid Ltd./Thermo Fisher Scientific, Basingstoke, UK) with 5% horse blood (Oxoid, Basingstoke, UK) and were incubated in anaerobic atmosphere (AnaeroGen sachets, Oxoid) at 37 °C for 2 to 7 days and up to 14 days for the sample of prosthetic joint infection. *C. acnes* strains were identified by routine methods such as Gram stain, colonial morphology, aerobic control, catalase, spot indole and API system RapID™ ANA II System (Oxoid) or MALDI TOF MS (VITEK^®^ MS, bioMérieux, Marcy l’Étoile, France).

Strain susceptibility was tested by E tests (E test strips, Liofilchem, Roseto degli Abruzzi, Italy) or breakpoint susceptibility test, as described in a previous publication, and disk diffusion method according to the EUCAST version 15.0, valid from 1 January 2025 [9,10,11]. Breakpoint susceptibility testing was performed by an agar dilution method with several antibiotic concentrations corresponding to susceptibility and resistance breakpoints [9].

Fastidious anaerobe agar supplemented with 5% defibrinated horse blood has been recommended for susceptibility testing of anaerobes, including minimal inhibitory concentration (MIC) determination and disk diffusion method [12]. We used Anaerobe basal agar (Oxoid) with 5% defibrinated horse blood (Oxoid), since the agar has a similar composition and is also supplemented by starch, hemin and vitamin K, sodium succinate, sodium pyruvate, arginine and L-cysteine hydrochloride.

Susceptibility to amoxicillin, clindamycin, tetracycline, levofloxacin, vancomycin and piperacillin/tazobactam was tested. Since not all strains from older years were available, testing with the disk diffusion method was performed on a smaller number of strains.

According to the EUCAST version 15.0, valid from 1 January 2025, resistance breakpoints were >0.25 mg/L for amoxicillin, <27 mm inhibitory zone for piperacillin/tazobactam 30–6 µg/disk, and >2 mg/L or <22 mm inhibitory zone for vancomycin 5 µg/disk [11].

Initially, all strains were tested for clindamycin resistance with a resistance breakpoint of >2 mg/L. Most strains (61) were recently retested according to EUCAST recommendations, and strains exhibiting MICs >0.25 mg/L clindamycin or inhibitory zones of <26 mm by 2 μg/disk clindamycin were considered as resistant to the agent [11]. There are no breakpoints for *C. acnes* to some antibiotics; therefore, we considered strains with minimal inhibitory concentrations (MICs) of >1 mg/L as resistant to tetracycline and levofloxacin. Metronidazole susceptibility is not included, since all *C. acnes* strains were resistant to the agent, and no metronidazole susceptibility testing is recommended for *C. acnes* in the current EUCAST [11].

### Statistical Analysis

Statistical analysis between groups was performed by chi-square with Fisher’s exact test (https://www.graphpad.com/quickcalcs/contingency1/ (accessed on 21 August 2025). Statistical differences of <0.05 were considered as significant.

## 3. Results

### 3.1. Phylotypes

The most common phylotypes among all strains are IA1 and IA2, accounting for 78.1% (86.3%, 58/73 isolates, Table 2).

By frequency, the phylotypes in the 55 acneic strains were phylotype I (90.9%, no. = 50 strains), including IA1 (61.8%, no. = 34 strains), and IA2, (21.8%, no. = 12 strains), II (7.3%, no. = 4), IB (5.4%, no. = 3), IA1 + II (1.8%, no. = 1), and III (1.8%, no. = 1).

The phylotypes in the 18 non-acneic strains, in decreasing order, were phylotype I (72.2%, no. = 13 strains), including IA1 (38.9%, no. = 7 strains), II (27.8%, no. = 5), and IA2 and IB (16.7% each, no. = 3 and 3). Phylotype II was >3-fold more frequent in non-acneic strains (27.8%) than in the acneic isolates (7.3%, *p* = 0.036), (Table 2).

### 3.2. Antibiotic Resistance

Resistance rates in acne-associated strains were >41% for clindamycin (45.4% by MICs and 41.9% by DDM), 36.4% for tetracycline and 15.9% for levofloxacin. Resistance rates in non-acneic clinical strains were >38% (38.8% by MICs and 38.9% by DDM) for clindamycin, 22.2% for tetracycline and 5.6% for levofloxacin. None of the 51 strains (33 acneic and 18 non-acneic strains) tested for susceptibility to piperacillin/tazobactam and vancomycin by DDM was found to be resistant (Table 3).

Three acneic strains (5.4%, 3/55 strains) and two non-acneic strains (11.1%, 2/18) exhibited resistance to amoxicillin.

There were no statistically significant differences (*p* ≥ 0.372) between resistance rates in acneic and non-acneic strains.

According to the phylotypes, amoxicillin resistance was 1.8% in I phylotype strains (2.4% in IA1 and 0.0% in IA2 strains) versus 23.5% in the non-I phylotype isolates (*p* = 0.009), (Table 4). Clindamycin resistance in IA1 phylotype was about 42% by both MIC and DDM and 52.9% (9/17) in non-I phylotype isolates. Tetracycline resistance (>1 mg/L) in IA1, IA2 and other strains was 36.6%, 33.3% and 23.5%, respectively. As for levofloxacin resistance (>1 mg/L), 16.7% of IA1 strains, 10.0% of IA2 strains and 6.2% of other phylotype strains were resistant to the agent.

### 3.3. Double and Multidrug Resistance (MDR)

Double resistance was found in 32.6% (14/43) acneic strains and in 22.2% (4/18, *p* = 0.544) non-acneic strains tested for susceptibility to all four antibiotics (amoxicillin, clindamycin, tetracycline and levofloxacin) to which resistance was detected (Table 5). The most frequent double resistance in acneic strains was to both tetracycline and levofloxacin in 18.6% (8/43) of the strains.

Double resistance to clindamycin and tetracycline was present in three acneic strains (7.0%) and in two non-acneic strains (11.0%). Amoxicillin + clindamycin resistance was detected in single (one acneic and two non-acneic) strains, and clindamycin + levofloxacin resistance was found in only one strain. Double resistance was present in different phenotypes except for phenotype III, which was only one strain.

The overall multidrug resistance rate in acneic strains was 9.3% (4/43 strains) but was not detected in the non-acneic strains. Multidrug resistance was found to amoxicillin, clindamycin and tetracycline in three strains and to clindamycin, tetracycline and levofloxacin in one strain. The phylotypes exhibiting triple resistance were IA2 (two strains), IA1 (one strain) and II (one strain).

## 4. Discussion

### 4.1. C. acnes Phylotypes

In China, Zhang et al. [13] evaluated *C. acnes* phylotypes from 63 acneic patients and found IA1 to be the prevalent phylotype (71.4%), followed in decreasing order by IA2 (19.0%), II (4.8%), IB (3.2%) and IC (1.6%), whereas phylotype III was not found. Similarly, in the present study, the predominant phylotype in acneic strains was IA1 (61.8%), followed in decreasing order by IA2 (21.8%), II (7.3%) and IB (5.4%). However, we also detected a mixed IA1 + II case (1.8%) and found phylotype III in one strain (1.8%).

It is difficult to distinguish between clinically significant *C. acnes* isolates in deep-seated infections and contaminants. Both et al. [14] found that almost all phylotypes can become invasive. The authors [14] suggested the presence of several positive specimens per patient or the presence of at least 10 colony-forming units per agar Petri dish to be useful for distinguishing the invasive strains from contaminants. We did not receive several clinical samples from all patients, but we took into account the condition of the presence of multiple colonies on the agar plate.

In the present study, among non-acneic strains, most of which were orthopedic isolates, I phylotype (72.2%) and mostly IA1 type (38.9%) were predominant. Phylotypes IA2 and IB were found in 16.7% each. Both et al. [14] have also reported IA1 phylotype to be the most prevalent type (48.3%) in deep-seated infections. Interestingly, in our study, phylotype II strains (27.8%) were 3.8-fold more frequent in non-acneic strains compared to the acneic strains (7.3%, *p* = 0.036). Although *C. acnes* subsp. *defendens* (type II) exhibits anti-inflammatory properties, some strains can also be associated with opportunistic infections. For instance, the type *C. acnes* subspecies *defendens* strain was from a subcutaneous abscess [15]. Both et al. [14] have also have reported that phylotype IA1 along with clonal complex CC30 (phylotype II) was significantly linked to deep-seated infections. Phylotype II was found in implant-associated infections by Aubin et al. [16] and in disk degeneration conditions by Capoor et al. [17] and Lin et al. [18].

In our study, phylotype III was not detected among the non-acneic strains. The result corresponds to the findings of Both et al. [14], who did not find such isolates in deep-seated infections.

### 4.2. C. acnes Resistance to Antibiotics

It is usually thought that *C. acnes* strains are uniformly susceptible to beta-lactams. Zhang et al. [13] did not detect ampicillin resistance (>0.25 mg/L) among 63 acneic strains of *C. acnes* in China in 2016–2017. There are relatively few studies reporting amoxicillin susceptibility testing for *C. acnes*. However, beta-lactam resistance was found in a few studies. Penicillin resistance (>0.5 mg/L) has been reported in 1 (4%) of 28 orthopedic isolates from the shoulder in the U.S. study of Crane et al. [19]. In *Cutibacterium* isolates in a Malaysian tertiary hospital, penicillin resistance has been reported in 18.7% (6/32 isolates) using CLSI M100 breakpoints [20]. Although, in a study in a tertiary-care hospital in Greece, no resistance (MIC > 0.5 mg/L) to penicillin and ampicillin was found in non-acneic *C. acnes* isolates associated with infections [21], in another Greek study, Grech [22] detected amoxicillin resistance in 4.4% among 90 acneic *C. acnes* isolates using a resistance breakpoint of 4 mg/L. This breakpoint is 16-fold higher than that used in our study (0.25 mg/L) according to the EUCAST [11]. This implies that *C. acnes* susceptibility to beta-lactams should be periodically monitored.

Importantly, amoxicillin resistance was 13-fold more frequent in the non-I phylotype isolates compared to phylotype I (IA1 + IA2) isolates. This can be explained by the usually more frequent use of beta-lactams to treat non-acneic versus acneic infections.

In Greek non-acneic isolates, clindamycin resistance was low (10% at MICs > 4 mg/L) [21]. Similarly, in Malaysia, only 1 (3.1%) of 32 hospital *Cutibacterium* isolates was clindamycin resistant according to CLSI M100 breakpoints [20]. We found a high (>43%) resistance rate to clindamycin in acneic strains and >38% in non-acneic strains by both BST and DDM. Our results were similar to those (about 40% of clindamycin resistance) reported by Aoki et al. [23] in Japan. Overall, tetracycline resistance was found in around 1/3 of the strains. On the contrary, overall levofloxacin resistance was much lower (12.9%) and only 15.9% in the acneic strains, probably because, unlike clindamycin and tetracycline, fluoroquinolones are not drugs of choice for treating acne [24].

Compared to the resistance rates in the systematic review and meta-analysis of Beig et al. [24], encompassing 27 countries over a prolonged period of 40 years, our study revealed a higher resistance rate for clindamycin (>38% versus 20.5%) and tetracyclines (32.9% versus 17.1%) but lower resistance for levofloxacin (around 13% versus 42.8%) when EUCAST breakpoints were considered. However, the authors detected an increase in clindamycin resistance rates from 1983–2014 to 2015–2023 [24].

Prolonged antibiotic therapy may increase resistance rates in *C. acnes* and other species of the skin microbiota [2]. In the systemic review of Walsh et al. [25], clindamycin resistance was lower (≤20%) in Chile, Colombia and Japan and high (>60%) in Egypt, France and Spain, and tetracycline resistance rates also largely varied. The authors have recommended the use of oral antibiotics only for moderate or severe acne and always in combination with topical agents such as retinoid or benzoyl peroxide [25].

No resistance to piperacillin/tazobactam was found, and no vancomycin resistance was observed among the 51 isolates tested. The results suggest that these agents are suitable for treating deep-seated *C. acnes* infections.

Antibiotic resistance in *C. acnes* can be a concern for the choice of antibacterial therapy for acne vulgaris. In Colombia, Castellanos et al. [26] evaluated acneic *C. acnes* isolates and found 5.4% resistance to tetracycline and doxycycline and 0.8% to minocycline. However, in Japan, Aoki et al. [23] investigated 127 acneic strains from 212 patients between 2013 and 2018 and observed an increase in 16S rRNA mutations, leading to diminished tetracycline susceptibility from 1.4% to 12.1%. Nevertheless, the use of different breakpoints for non-susceptibility or resistance in *C. acnes* renders the comparison between results of different studies difficult.

Minocycline, and especially the new tetracycline antibiotic, sarecycline, have shown advantages over tetracycline and doxycycline for the treatment of acne vulgaris [27,28]. Sarecycline is a well-tolerated and narrow-spectrum derivative of tetracycline that can reduce inflammatory lesions in about 20 days and has shown a low in vitro development of resistance [28,29,30].

*C. acnes* resistance to antibiotics has most often been associated with chromosomal point mutations in the 23S rRNA gene for resistance to macrolides and in the 16S rRNA gene for tetracycline resistance, and resistance to both clindamycin and erythromycin has been linked to the *erm(X)* gene [2]. *C. acnes* resistance to fluoroquinolones is often due to mutations in the *gyrA* gene as well as to efflux pumps [31].

### 4.3. Multidrug Resistance

The presence of *C. acnes* strains with double or multidrug nonsusceptibility/resistance can strongly limit the choice of antibiotics for treatment of the associated infections. In Jordan, Alkhawaja et al. [32] found similar resistance patterns in multidrug resistant acneic *C. acnes* isolates and either *Staphylococcus aureus* or *Staphylococcus epidermidis* isolates in some patients. In Greece, Grech [22] detected acneic *C. acnes* strains resistant to all the antibiotics tested (amoxicillin, clindamycin, erythromycin and minocycline).

In the present study, double resistance was found in 29.5% of 61 strains tested for susceptibility to amoxicillin, clindamycin, tetracycline and levofloxacin, without statistical difference between the acneic (32.6%) and non-acneic strains (22.2%). The most common double resistance was to tetracycline and either levofloxacin or clindamycin, found in 13 strains. Although not detected in non-acneic strains, MDR was found in 9.3% of the acneic strains. Three strains were resistant to amoxicillin, clindamycin and tetracycline, and one strain was resistant to clindamycin, tetracycline and levofloxacin. Although, *C. acnes* resistance to antibiotics was most often linked to SPN polymorphisms in the 23S rRNA gene for lincosamides and macrolides and 16S rRNA gene for tetracyclines, and *gyrA* and *gyrB* mutations for fluoroquinolones, multidrug resistant plasmids, transposon acquisition and efflux pumps can cause MDR in *C. acnes* [5,6].

The results reveal the already unpredictable susceptibility patterns of *C. acnes* and imply the need of susceptibility testing of the individual isolates in order to improve therapeutic success. However, the vancomycin and piperacillin/tazobactam susceptibility of all strains, mainly from orthopedic infections, suggests their therapeutic benefits. In the review article of Bonnet and Lourtet-Hascoët, these two agents can be used even in combination for treating polymicrobial orthopedic infections, which can constitute up to 25% of orthopedic infections [33].

For the treatment of *C. acnes*-associated infections, susceptibility to lincosamides, tetracyclines and quinolones, including beta-lactams, should be monitored periodically, and it is best to test the susceptibility of individual isolates before choosing an antibiotic. Anti-biofilm agents, synthetic antimicrobial peptides and bacteriophages may also be implemented [33,34]; however, more studies are required to prove their benefits.

Strengths of the present study are the comparison of the results according to both strain origin and phylotypes. Limitations of the study are the relatively small group (only 18 strains) of non-acneic strains and the use of several susceptibility testing methods, alone or in combination, due to the recent suggestion of DDM by EUCAST, the change in resistance breakpoints over time, as well as the difference in the numbers of strains tested for some antibiotics due to loss of some strains over the years.

## 5. Conclusions

In conclusion, the IA1 phylotype was dominant in both acneic and non-acneic strains, and type II was more frequent in non-acneic isolates. Although, it is usually thought that *C. acnes* isolates remain fully susceptible to beta-lactams; however, our results revealed amoxicillin resistance in >6% of the strains, which represents a rare yet important finding. Amoxicillin resistance was higher in non-phylotype I strains. We detected high rates of clindamycin and tetracycline resistance and lower levofloxacin resistance rates. The strains were uniformly susceptible only to vancomycin and piperacillin/tazobactam, which confirms that these antibiotics, alone or in combination, are suitable for treating orthopedic infections. The resistance rates and the presence of double and multidrug resistance strongly imply the need of regular monitoring of *C. acnes* susceptibility to antibiotics and susceptibility-guided therapy of the associated infections such as acne vulgaris.

## Figures and Tables

**Table 1:** Patients’ characteristics included in the study.

Strains	Patients’ Characteristics	Specimens	No. of Patients
Acneic	Acne vulgaris	Pustule	55
	Children (aged 12–17 years)		10
	Adults (aged 17–50 years)		45
	Boys/men		15
	Girls/women		40
Non-acneic	Adults (aged 43–81 years)		18
	Men		6
	Women		12
	Diseases/conditions		
	Orthopedic		
	Wound	Wound	6
	Wound dehiscence	Wound	1
	Post-surgical wound	Wound	2
	Osteomyelitis	Biopsy	2
	Bone metastases	Punctate	1
	Mobilization of endoprosthesis	Punctate	2
	Purulent gonitis	Tissue	1
	Prosthetic joint infection	Tissue	1
	Ankle fracture	Wound	1
	Other		
	Rosacea	Pustule	1

**Table 2:** Phylotypes of *C. acnes* in acneic and non-acneic strains.

Phylotypes	In Acneic Strains (No. = 55)	% of Acneic Strains	In Non-Acneic Strains (No. = 18)	% of Non-Acneic Strains	*p* Value	Total (No. = 73)	Total %
IA1	34	61.8	7	38.9	0.107	41	56.2
IA2	12	21.8	3	16.7	0.748	15	20.5
IB	3	5.4	3	16.7	0.155	6	8.2
IA1 + II	1	1.8	0	0.0	1.000	1	1.4
I (total)	50	90.9	13	72.2	0.107	63	86.3
II	4	7.3	5	27.8	0.036	9	12.3
III	1	1.8	0	0.0	1.000	1	1.4

**Table 3:** *C. acnes* resistance to antibiotics according to the type of the patients.

Antibiotic/Resistance Breakpoint (mg/L or mm)	Type of Strains	No. of Strains Tested	Methods (No. of Strains Tested)	No. of Resistant Strains	% of Resistance **
Amoxicillin (>0.25 mg/L *)	Acneic	55	Et (34), BST (21)	3	5.4
	Non-acneic	18	Et (13), BST (5)	2	11.1
	Total	73	Et (47), BST (26)	5	6.8
Clindamycin (>2 mg/L)	Acneic	55	Et (24) or BST (31)	25	45.4
	Non-acneic	18	Et (4) or BST (14)	7	38.8
	Total	73	Et (28) or BST (45)	32	43.8
Clindamycin (<26 mm *)	Acneic	43	DDM (2 µg/disk)	18	41.9
	Non-acneic	18	DDM (2 µg/disk)	7	38.9
	Total	61	DDM (2 µg/disk)	25	41.0
Tetracycline (>1 mg/L)	Acneic	55	Et (27) or BST (28)	20	36.4
	Non-acneic	18	Et (10) or BST (8)	4	22.2
	Total	73	Et (37) or BST (36)	24	32.9
Levofloxacin (>1 mg/L)	Acneic	44	Et (33)	7	15.9
	Non-acneic	18	Et (18)	1	5.6
	Total	62	Et (51)	8	12.9
Piperacillin/ tazobactam (<27 mm *)	Acneic	33	DDM (30/6 µg/disk)	0	0
	Non-acneic	18	DDM (30/6 µg/disk)	0	0
	Total	51	DDM (30/6 µg/disk)	0	0
Vancomycin (<22 mm *)	Acneic	33	DDM (5 µg/disk)	0	0
	Non-acneic	18	DDM (5 µg/disk)	0	0
	Total	51	DDM (5 µg/disk)	0	0

Legend: Et—E test; BST—breakpoint susceptibility testing; DDM—disk diffusion method; * EUCAST—The European Committee on Antimicrobial Susceptibility Testing. Breakpoint tables for interpretation of MICs and zone diameters. Version 15.0, 2025. https://www.eucast.org (accessed on 27 July 2025). ** the statistical differences between resistance rates in acneic and non-acneic isolates were not significant (*p* > 0.388).

**Table 4:** *C. acnes* resistance to antibiotics according to the phylotypes.

Antibiotic	Phylotype	No. of Strains Tested **	No. of Resistant	% of Resistance
Amoxicillin	IA1	41	1	2.4
	IA2	15	0	0.0
	IA1 + IA2	56	1	1.8 *
	others	17	4	23.5 *
	Total	73	5	6.8
Clindamycin >2 mg/L	IA1	41	17	41.5
	IA2	15	6	40.0
	IA1 + IA2	56	23	41.1
	Others	17	9	52.9
	Total	73	32	43.8
Clindamycin DDM	IA1	36	15	41.7
	IA2	11	4	36.4
	IA1 + IA2	47	19	40.4
	Others	14	6	42.8
	Total	61	25	41.0
Tetracycline	IA1	41	15	36.6
	IA2	15	5	33.3
	IA1 + IA2	56	20	35.7
	Others	17	4	23.5
	Total	73	24	32.9
Levofloxacin	IA1	36	6	16.7
	IA2	10	1	10.0
	IA1 + IA2	46	7	15.2
	Others	16	1	6.2
	Total	62	8	12.9

Legend: * Statistically significant (*p* = 0.009); ** the methods used are presented in Table 2.

**Table 5:** Double and multidrug resistance.

Strains *	Acneic	Non-Acneic	Total
No. of strains tested	43	18	61
Double resistance (No.)	14	4	18
Double resistance (%)	32.6	22.2	29.5
Double resistance to	TET + LFX (8 strains),		TET + LFX (8 strains),
	CLI + TET (3 strains),	CLI + TET (2 strains),	CLI + TET (5 strains),
	CLI + LFX (2 strains),		CLI + LFX (2 strains),
	AMX + CLI (1 strain)	AMX + CLI (2 strains)	AMX + CLI (3 strains)
Double-resistant phylotypes	IA1 (8 strains),	IA1 (3 strains), IB (1 strain)	IA1 (11 strains),
	IA2 (3 strains),		IA2 (3 strains),
	IB (1 strain),		IB (2 strain),
	II (1 strain),		II (1 strain),
	IA1 + II (1 strain)		IA1 + II (1 strain)
MDR resistance (No.)	4	0	4
MDR resistance (%)	9.3	0.0	6.6
MDR resistance to	AMX + CLI + TET (3 strains)	0.0	AMX + CLI + TET (3 strains)
	CLI + TET + LFX (1 strain)		CLI + TET + LFX (1 strain)
MDR phylotypes	IA1 (1 strain),	None	IA1 (1 strain),
	IA2 (2 strains),		IA2 (2 strains),
	II (1 strain)		II (1 strain)

Legend: * Only the strains tested for amoxicillin, clindamycin, tetracycline and levofloxacin susceptibility. AMX—amoxicillin; CLI—clindamycin; MDR—multidrug resistance; TET—tetracycline; LFX—levofloxacin.

## Data Availability

The original contributions presented in this study are included in the article. Further inquiries can be directed to the corresponding author.
